# Blood Cell-Bound C4d as a Marker of Complement Activation in Patients With the Antiphospholipid Syndrome

**DOI:** 10.3389/fimmu.2019.00773

**Published:** 2019-04-12

**Authors:** Paola Adele Lonati, Mariangela Scavone, Maria Gerosa, Maria Orietta Borghi, Francesca Pregnolato, Daniele Curreli, Gianmarco Podda, Eti Alessandra Femia, Wilma Barcellini, Marco Cattaneo, Francesco Tedesco, Pier Luigi Meroni

**Affiliations:** ^1^Immunorheumatology Research Laboratory, Istituto Auxologico Italiano, IRCCS, Milan, Italy; ^2^Unità di Medicina II, ASST Santi Paolo e Carlo, Milan, Italy; ^3^Dipartimento di Scienze Della Salute, University of Milan, Milan, Italy; ^4^Dipartimento di Scienze Cliniche e di Comunità, University of Milan, Milan, Italy; ^5^UOC Ematologia, Fondazione IRCCS Ca' Granda Ospedale Maggiore Policlinico, Milan, Italy

**Keywords:** anti-phospholipid syndrome, beta2–glycoprotein I, complement, C4d, platelets, erythrocytes

## Abstract

Antiphospholipid syndrome (APS) is a chronic and disabling condition characterized by recurrent thrombosis and miscarriages mediated by antibodies against phospholipid-binding proteins (aPL), such as beta_2_glycoprotein I (β_2_GPI). Complement is involved in APS animal models and complement deposits have been documented in placenta and thrombotic vessels despite normal serum levels. Analysis of circulating blood cells coated with C4d displays higher sensitivity than the conventional assays that measure soluble native complement components and their unstable activation products in systemic lupus erythematosus (SLE). As C4d-coated blood cell count has been reported to be more sensitive than serum levels of complement components and their activation products in systemic lupus erythematosus (SLE) patients, we decided to evaluate the percentage of C4d positive B lymphocytes (BC4d), erythrocytes (EC4d), and platelets (PC4d) in primary APS patients and asymptomatic aPL positive carriers as marker of complement activation in APS. We assessed by flow cytometry the percentages of BC4d, EC4d, and PC4d in primary APS (PAPS; n. 23), 8 asymptomatic aPL positive carriers, 11 APS-associated SLE (SAPS), 17 aPL positive SLE, 16 aPL negative SLE, 8 aPL negative patients with previous thrombosis, 11 immune thrombocytopenia (ITP) patients, and 26 healthy subjects. In addition, we used an *in vitro* model to evaluate the ability of a monoclonal anti-β_2_GPI antibody (MBB2) to bind to normal resting or activated platelets and fix complement. EC4d and PC4d percentages were significantly higher in PAPS and aPL carriers as well as aPL positive SLE and SAPS than in aPL negative controls. The highest values were found in PAPS and in SAPS. The EC4d and PC4d percentages were significantly correlated with serum C3/C4 and anti-β_2_GPI/anti-cardiolipin IgG. *In vitro* studies showed that MBB2 bound to activated platelets only and induced C4d deposition. The detection of the activation product C4d on circulating erythrocytes and platelets supports the role of complement activation in APS. Complement may represent a new therapeutic target for better treatment and prevention of disability of APS patients.

## Introduction

Antiphospholipid syndrome (APS) is a chronic autoimmune disease characterized by recurrent thrombotic events and pregnancy morbidity, in the presence of antibodies targeting anti-phospholipid binding proteins (aPL) (1). APS can be found in patients with no evidence of associated diseases (primary APS, PAPS) or with other autoimmune disorders, mainly systemic lupus erythematosus (SLE) (SAPS) (1).

The major aPL antigenic target is β_2_-glycoprotein I (β_2_GPI), a phospholipid binding plasma protein of 50 kDa (2). Upon binding to anionic phospholipids or to membrane receptors of different cell types, β_2_GPI changes conformation, exposing an immunodominant cryptic epitope located at domain I (D1), which is recognized by aPL. There is sound evidence from *in vitro* and *in vivo* models that β_2_GPI-dependent aPL play a pathogenic role both in thrombosis and pregnancy complications (3, 4). Moreover, epidemiological data support a strong diagnostic/prognostic value of anti-D1 antibodies in APS patients (5).

Complement activation was initially suggested to be involved in APS animal models since the induction of fetal loss or thrombosis by passive infusion of aPL IgG was prevented by treatment with inhibitors of complement activation or the use of animals deficient in complement components (6–10). Moreover, a human monoclonal antibody against β_2_GPI D1 lacking the complement-fixing portion of the molecule (MBB2ΔCH2), unlike the complement-fixing parent molecule (MBB2) that reacts with the same epitope (11) fails to exhibit pathogenic effect. In contrast, low C3 and C4 serum levels were described in some APS patients only and few studies reported high levels of complement activation products (fragment Bb and anaphylatoxins C4a, C3a, and C5a) with no association with the vascular manifestations of the syndrome (12–15). On the other hand, we recently reported deposition of C1q, C4, C3, and C9 on the endothelium of the vessel wall close to the thrombotic occlusion in a PAPS patient who underwent bypass surgery to treat an arterial thrombotic occlusion. Notably, complement components co-localized with β_2_GPI and IgG, suggesting that aPL caused complement activation and contributed to the pathogenesis of the thrombotic event (16).

Measurement of serum levels of complement activation products has been reported to be more sensitive than that of native complement components in SLE (17). In particular, the number of C4d-coated B lymphocytes, erythrocytes and platelets in circulating blood of SLE patients with active disease was higher than in controls (17–24).

The number of C4d-bound platelets was associated with lupus disease activity and complement consumption but contrasting results regarding the association with arterial or venous events and aPL were reported (25, 26). This finding is in contrast with the ability of aPL to activate complement and promote binding of complement split products to fixed platelets *in vitro* (25, 27, 28).

We have investigated the percentage of C4d positive circulating blood cells in PAPS and report a higher number of C4d positive erythrocytes and platelets in aPL positive patients than in controls supporting the hypothesis that complement is activated *in vivo*. A mechanistic model of complement deposition on platelets in the presence of a human monoclonal antibody specific for D1 of β_2_GPI was investigated as well.

## Materials and Methods

### Patients and Controls

We recruited the following patients: 23 PAPS; 11 SAPS, 17 aPL positive SLE, and 16 aPL negative SLE. As controls we included: 11 patients with primary immune thrombocytopenia (ITP), 8 aPL negative patients with previous thrombotic episodes, 8 persistently positive aPL healthy subjects (aPL positive carriers), and 26 normal healthy subjects (NHS). SLE patients were classified according to the 1997 American College of Rheumatology updated criteria for SLE and the new 2012 SLICC SLE criteria (29, 30). Disease activity in SLE patients was assessed by the SELENA version of SLEDAI index in SLE (31). APS patients fulfilled the revised Sapporo criteria (32). The characterization of vascular and obstetric APS was carried out as previously reported (33). Primary ITP was diagnosed according to the International Working Group criteria (34).

All ITP patients were in stable clinical remission. Anti-phospholipid negative patients with thrombosis had previous documented episodes of myocardial infarction, stroke, transient ischemic attack, pulmonary embolism, or deep vein thrombosis.

When entering the protocol, all subjects underwent a detailed clinical evaluation and comprehensive medical history registration. Recorded variables included sex, age, disease duration and therapy (Supplementary Table 1). To avoid any modification due to the acute phase reactants, samples have been collected at least six months after the thrombotic event (35). The protocol was approved by the local Institutional Review Board (IRB) (Comitato Etico Milano area 2–426_2014) and all subjects gave written informed consent.

### Blood Samples

Venous blood samples were collected from an antecubital vein using a 21-gauge butterfly needle with minimal stasis. The first 3 mL were collected into K2E EDTA tubes (Becton Dickinson vacutainer, North Ryde, NSW, Australia) and analyzed by coulter hematology analyser for blood cell count (DxH-800, Beckman Coulter, Milano, Italy). Additional blood samples included: 3 mL in K2E EDTA for measurement of C4d deposition on B lymphocytes, red blood cells and platelets by flow cytometry; 3 mL in CTAD vacutainer for lupus anticoagulant (LAC) determination; 5 mL in vacutainer without anticoagulant for serum collection. Five mL of blood were collected into desirudin (Revasc, 25 μg/mL) and used for experiments of C4d deposition on *in vitro* stimulated and resting platelets.

### Serum Complement Determination

Serum concentrations of complement components C3 and C4 were determined by an immunoturbidimetric method (Roche/Hitachi cobas c 701/702): C3 and C4 normal ranges indicated by the manufacturer were 55–180 and 20–50 mg/dL, respectively.

### Detection of aPL

Serum anticardiolipin (anti-CL) and anti-β_2_GPI IgG/IgM autoantibodies were detected as previously described (36). LAC was measured according to international ISTH guidelines (37).

### Detection of C4d Bound to Cells by Flow Cytometry

The percentage of C4d bound to B lymphocytes (BC4d), erythrocytes (EC4d), and platelets (PC4d) was measured by flow cytometry after subtraction of background signals. All analyses were performed using a FACS Calibur cytometer and Cell Quest software (BD Biosciences, San Jose, CA). In all experiments, control procedures to establish proper calibration, compensation, and linearity were performed.

### C4d Bound to B-Lymphocytes

Erythrocytes lysis was performed by addition of ammonium chloride–based reagent (BD Pharm Lyse; BD Biosciences, San Jose, CA) to EDTA-whole blood (300 μL), left 10 min at 4°C and centrifuged at 800 g at 4°C for 5 min. Cell pellet was suspended in 1 mL of Dulbecco's phosphate buffered saline (DPBS, Sigma-Aldrich, St. Louis, MO) supplemented with 1% heat-inactivated fetal calf serum (FCS) (Sigma-Aldrich, St. Louis, MO) (DPBS/1% FCS) and stained at 2–8°C with 10 μg/mL purified mouse monoclonal antibodies against human C4d (mouse anti-human C4d; Quidel, San Diego, CA) or 10 μg/mL mouse anti-human isotype control IgG1κ (MOPC-21; BD Biosciences, San Jose, CA) for 45 min. Samples were then washed with DPBS/1%FCS and centrifuged at 800 g at 4°C for 5 min. Pellets were suspended in a DPBS/1%FCS solution containing fluorescein isothiocyanate (FITC) conjugated goat anti-mouse antibody (Cappel, MP biomedicals, Santa Ana, CA) (10 μg/mL) and R-phycoerythrin (PE)-conjugated monoclonal antibody against human CD-19 (a 95-Kd type I transmembrane glycoprotein expressed on B cells) (BD Biosciences, San Jose, CA), and stained at 2–8°C in the dark for 45 min. Samples were washed again and suspended in 250 μL cold DPBS/1%FCS. 5,000 events in the lymphocytes gate (based on their size) were acquired at high flow rate. A single fluorochrome dot plot strategy was used to identify B-lymphocytes CD19 positive (SSC vs. CD19-PE) and the percentage of C4d was assessed on B-lymphocytes gate (SSC vs. FITC).

### C4d Bound to Erythrocytes

EDTA-whole blood (50 μL) was suspended in 1,5 mL DPBS/1%FCS and centrifuged for 5 min at 800 g at 4°C. The pellet was suspended in 500 μL DPBS/1%FCS; 10 μL of the suspension was subsequently stained with anti-C4d monoclonal antibody or mouse anti-human isotype control IgG1κ at 2–8°C for 45 min. Samples were then washed and cell surface C4d was detected by addition of FITC-conjugated goat anti-mouse antibody at 2–8°C in the dark for 45 min. Samples were washed again and suspended in 250 μL of cold DPBS/1%FCS. 5,000 events in the erythrocytes gate were acquired with a high flow rate. A single fluorochrome dot plot strategy (FSC vs. FITC) was used for quantification of percentage of C4d on erythrocytes. Details regarding the erythrocyte gating strategy are shown in Supplementary Figure 1.

### C4d Bound to Resting Platelets

EDTA-whole blood samples (50 μL) were diluted with DPBS/1%FCS (1:2) and stained with anti-C4d monoclonal antibody or mouse anti-human isotype control IgG1κ at 2–8°C for 45 min, followed by staining with FITC-conjugated goat anti-mouse antibody at 2–8°C in the dark for 45 min. A PE–conjugated monoclonal antibody against human CD42b (a 145 kD glycoprotein known as GPIbα) was used to identify platelets. 5,000 events in the platelet gate were acquired at low flow rate. A single fluorochrome dot plot strategy was used to identify CD42b positive platelets (SSC vs. CD42b-PE) and the percentage of C4d was measured (SSC vs. FITC). Details regarding the platelet gating strategy are shown in Supplementary Figure 2.

### MBB2 Antibody

The human monoclonal antibody against D1 of β_2_GPI (MBB2) has been produced and characterized as previously published (11).

### *In vitro* Model of C4d Binding to Normal Platelets

Hirudin-anticoagulated whole blood from healthy blood donors was diluted in home-made PBS (1:15) and incubated with or without 500 μg/mL of MBB2 and/or thrombin receptor activating peptide (TRAP, Sigma-Aldrich, Germany) (20 μM) at 37°C for 20 min. Samples were then diluted with PBS (1:10) and stained with 10 μg/mL mouse anti-human C4d or 10 μg/mL mouse anti-human isotype control IgG1κ at RT for 20 min. Then, FITC-conjugated goat anti-human antibody (Cappel, MP biomedicals, Santa Ana, CA) and/or APC-conjugated goat anti-mouse antibody (ThermoFisher scientific Massachusetts, USA), and/or anti-CD42b-PE, and/or anti-CD62p-Pe/Cy7 (Biolegend, San Diego, CA) were added to the samples and incubated at RT in the dark for 20 min. After incubation, samples were diluted in 300 μL PBS and immediately analyzed by flow cytometry. All analyses were performed using a FACS Verse cytometer and FACS Suite software (BD Biosciences, San Jose, CA). 5,000 events in the platelet gate were acquired with a medium flow rate. Dot plot strategy was used for analysis: first of all, platelets were identified with CD42b-PE vs. SSC, then C4d-APC or MBB2-FITC or CD62p-Pe/Cy7 vs. SSC were used to determine the percentage of C4d and MBB2 platelet bound and of CD62p expression.

### Statistical Analysis

Statistical analysis was performed using GraphPad Prism version 6.0 (GraphPad Software Inc., San Diego, CA, USA). All results were expressed as median with interquartile range. The normality of data was evaluated by the D'Agostino-Pearson test. Comparisons between groups were performed using Kruskal-Wallis or Friedman tests as appropriate followed by Dunn's *post-hoc* test. Comparison between two groups were performed using Wilcoxon *t*-test. All tests were two tailed. Correlations between variables were expressed as Spearman's correlation coefficient. *p* < 0.05 was chosen as the cut-off level for statistical significance.

## Results

### aPL Profile of Patients and Controls

Normal healthy subjects and pathological controls (ITP, SLE, and patients with previous thrombosis) were negative in all aPL assays. Among the 17 aPL positive SLE patients, three displayed single, 7 double and 7 triple positivity. Four out of 11 SAPS patients were single-positive, 1 double-positive and 6 triple-positive for aPL; 4/8 aPL positive carriers were double- and 4/8 triple-positive; of the 23 PAPS patients, 1 was single-, 5 double-, and 17 triple-positive.

### C3 and C4 Serum Levels

aPL positive carriers and PAPS patients displayed lower serum levels of C3 and C4 than normal healthy subjects, albeit their values fell in the normal range (Table 1).

**Table 1:** Serum levels of C3 and C4.

	**NHS (*n* = 26)**	**ITP (*n* = 11)**	**aPL neg thrombosis (*n* = 8)**	**aPL neg SLE (*n* = 16)**	**aPL pos SLE (*n* = 17)**	**SAPS (*n* = 11)**	**aPL pos carriers (*n* = 8)**	**PAPS (*n* = 23)**
C3 (mg/dL) (normal range: 55–180 mg/dL)	129 ± 9	124 ± 11	159 ± 11	92 ± 6	76 ± 5	73 ± 7	97 ± 11	89 ± 4
C4 (mg/dL) (normal range: 20–50 mg/dL)	31 ± 3	25 ± 3	34 ± 4	19 ± 3	10 ± 1	15 ± 4	20 ± 2	20 ± 2

*NHS, normal healthy subjects; ITP, primary immune thrombocytopenia; aPL, anti-phospholipid antibodies; SLE, systemic lupus erythematosus; SAPS, secondary antiphospholipid syndrome; PAPS, primary antiphospholipid syndrome*.

*Values are reported as mean ± SEM*.

### Measurement of Cell-Bound C4d

**Figure 1:**
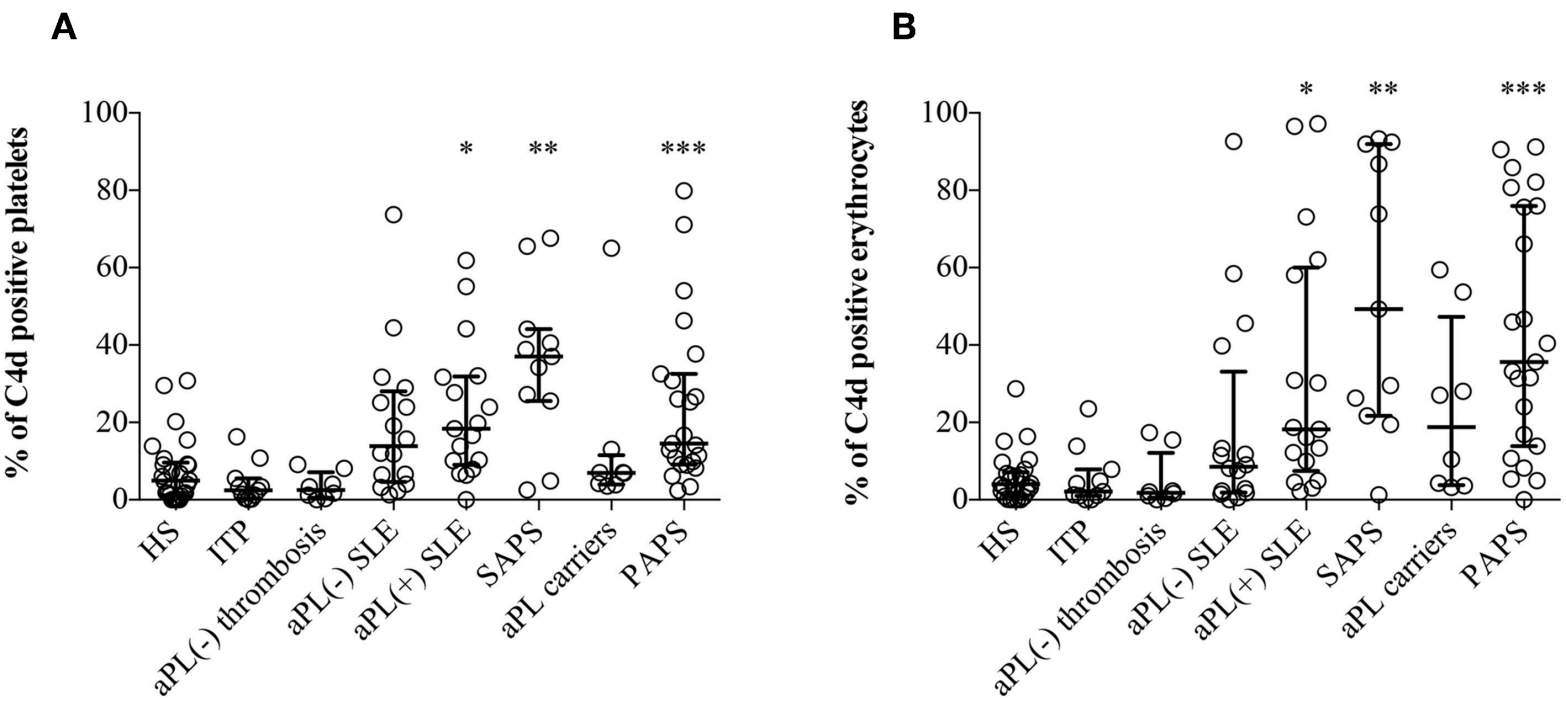
C4d deposition on platelets and erythrocytes. Flow cytometry was performed on EDTA-whole blood samples (*n* = 120). C4d-positive cells were detected by purified anti-human C4d and FITC conjugated goat anti-mouse antibody on platelets **(A)** and on erythrocytes **(B)**. Results are expressed as percentage, median with interquartile range, and analyzed by Kruskal-Wallis test and Dunn's multiple comparison *post-hoc* test. ^*^p < 0.05; ^**^p < 0.01, ^***^p < 0.001 vs. NHS.

Significantly higher percentages of both EC4d and PC4d were found in aPL positive SLE, SAPS and PAPS patients compared to NHS, ITP patients, and aPL negative thrombotic patients; moreover, higher percentages of EC4d and PC4d were detected also in aPL negative SLE and asymptomatic healthy aPL positive carriers (Figures 1A,B). Patients with SLE with or without aPL or full blown APS displayed higher BC4d percentages than PAPS or aPL positive carriers. On the contrary, the percentages of BC4d in aPL positive carriers and PAPS were comparable with the controls (Supplementary Figure 3).

Cell-bound C4d percentages were comparable both in thrombotic and obstetric APS patients, suggesting that they were not associated with the main APS clinical manifestations (Supplementary Figure 4); moreover, we did not find any correlation also with non-classification criteria (e.g., thrombocytopenia; Supplementary Figures 5–7) (32).

### Correlations Between the Percentage of PC4d and EC4d With Other Laboratory Parameters

**Figure 2:**
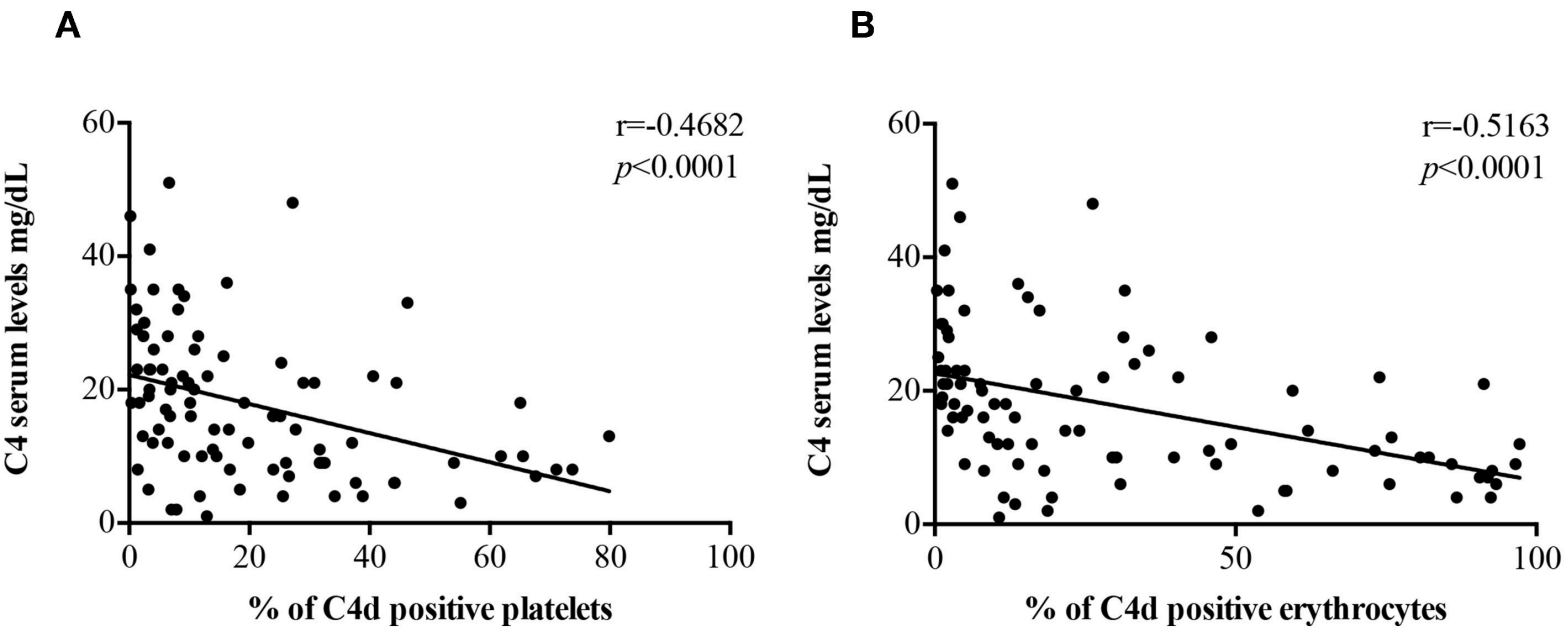
Correlations between C4 serum levels and EC4d and PC4d percentages. C4 serum levels (mg/dL) negatively correlated with the percentages of PC4d **(A)** and EC4d **(B)**.

C4 Serum levels were found to be inversely correlated with the percentage of PC4d (*r* = −0.4682, *p* < 0.0001; Figure 2A) and EC4d (*r* = −0.5163, *p* < 0.0001; Figure 2B).

**Figure 3:**
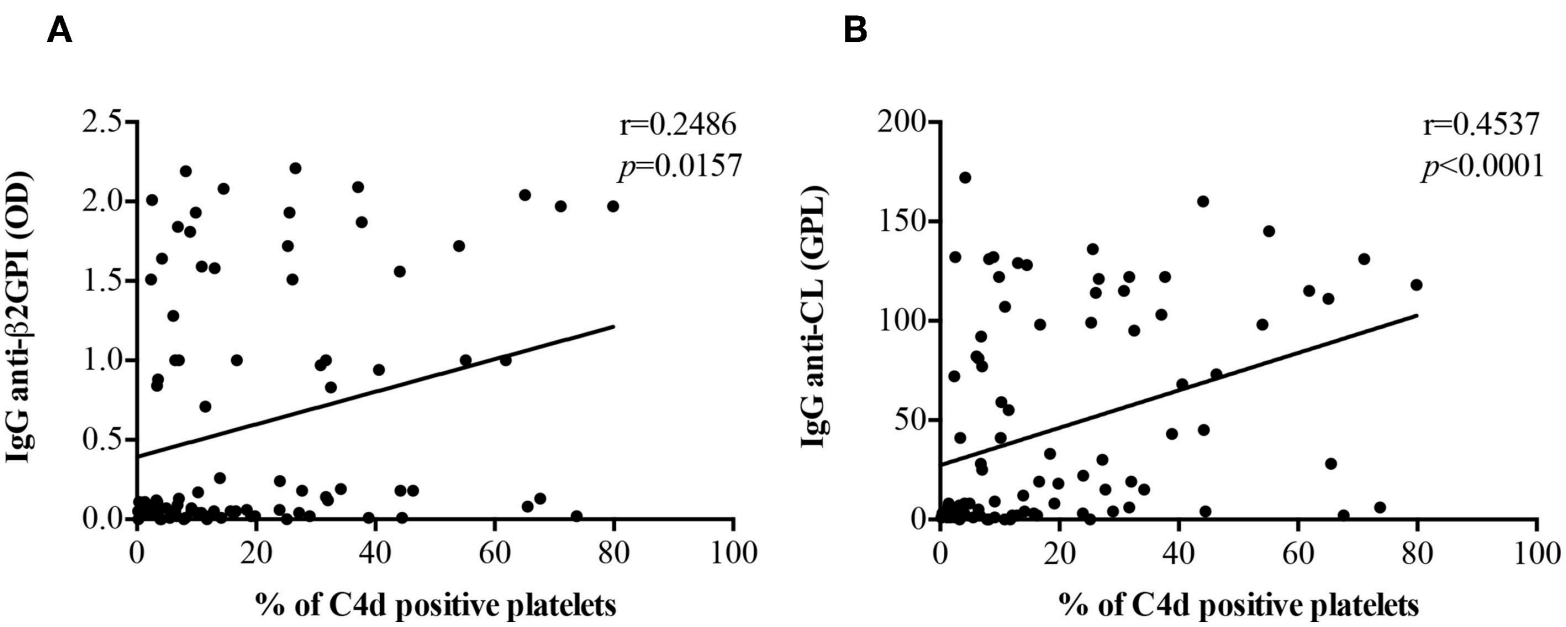
Correlations between anti-β2GPI and anti-CL IgG titers PC4d. PC4d titers positively correlated with both IgG anti-β_2_GPI **(A)** and IgG β_2_GPI-dependent anti-CL titers **(B)**.

There was a positive correlation of both anti-β_2_GPI IgG (*r* = 0.2486, *p* = 0.0157) and anti-CL IgG (*r* = 0.4537, *p* < 0.0001) titers with PC4d (Figures 3A,B). The correlation between PC4d and anti-CL IgM was mild, but statistically significant (*r* = 0.2472, *p* = 0.0163; Supplementary Figure 8B). In contrast, there was no correlation between PC4d and anti-β_2_GPI IgM titers (*r* = 0.09486, *p* = 0.3631) (Supplementary Figure 8A). EC4d percentages positively correlated with anti-β_2_GPI IgG (*r* = 0.3503, *p* = 0.0005) and anti-CL IgG (*r* = 0.4805, *p* < 0.0001) (Supplementary Figures 9A,B). There was also a positive correlation between EC4d and both anti-β_2_GPI IgM (*r* = 0.2672, *p* = 0.0092) and anti-CL IgM (*r* = 0.4177, *p* < 0.0001) (Supplementary Figures 9C,D).

### *In vitro* Studies: MBB2 and C4d Deposition on Resting and Activated Platelets

**Figure 4:**
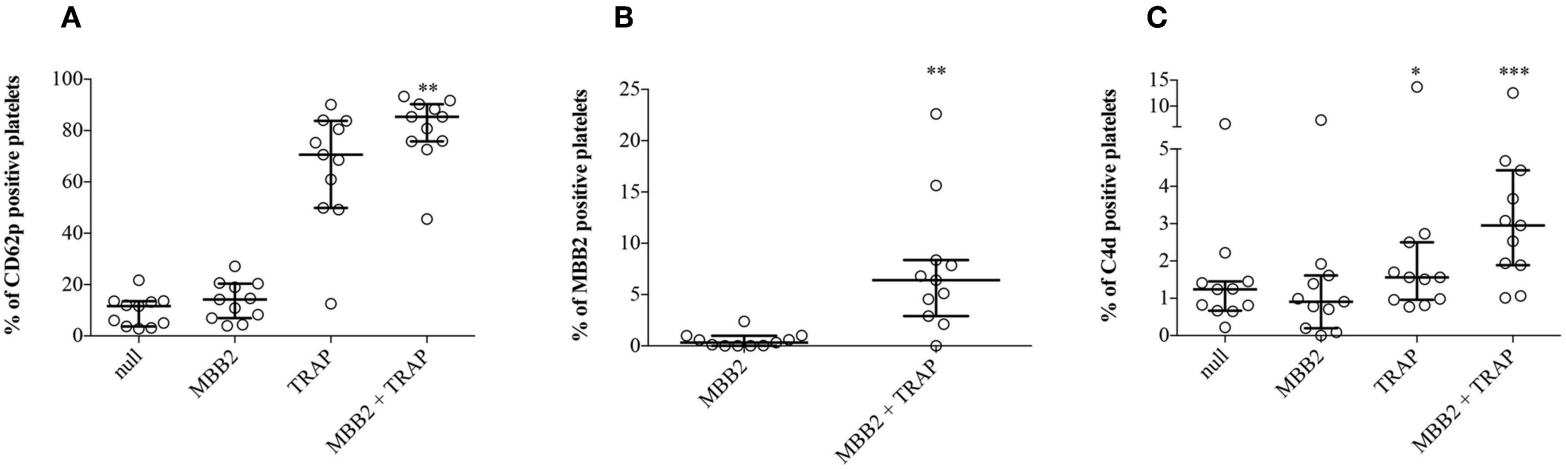
Effects of MBB2 and TRAP, alone and in combination, on the expression of CD62p on platelets. Percentages of CD62p-, MBB2-, and C4d-positive platelets were measured in hirudin-anticoagulated blood from 11 NHS after its incubation with or without MBB2 and/or TRAP at 37°C for 20 min. Data were analyzed by Friedman test and Dunn's multiple comparisons *post-hoc* test **(A,C)** and by Wilcoxon matched paired test **(B)**. Results are expressed as percentage, median with interquartile range. ^*^p < 0.05; ^**^p < 0.01, ^***^p < 0.001 vs. MBB2.

Since β_2_GPI-dependent aPL may recognize β_2_GPI adhered on activated platelets (3), we set up an *in vitro* model to investigate whether the bound antibody was responsible for cell membrane deposition of C4d. To this end, hirudin-anticoagulated whole blood samples from NHS (*n* = 11) were incubated with TRAP, a platelet agonist. In the absence of exogenous stimuli, the expression of CD62p on platelets was negligible, while it dramatically increased after addition of TRAP. These results indicate that circulating NHS platelets were not activated, offering a suitable *in vitro* model to investigate the possible role of platelet activation in β_2_GPI adhesion to cell membranes and antibody binding (Figure 4A). Blood samples, which provided the source of resting platelets, autologous β_2_GPI and complement, were incubated with MBB2. The binding of MBB2 to resting platelets was negligible (0.4%) but it increased up to 18 times (7.5%; *p* = 0.002) following platelet stimulation by TRAP (Figure 4B). This finding suggests that pathogenic aPL may recognize autologous β_2_GPI bound only to activated platelets. We then investigated whether the bound antibody was able to activate complement and to induce C4d deposition on the platelet membranes. The percentages of PC4d increased significantly when the blood samples were simultaneously incubated with TRAP and MBB2, suggesting that MBB2 reacted with adhered β_2_GPI and activated complement, leading to C4d deposition on activated platelets (Figure 4C).

## Discussion

Complement has been shown to be activated in several systemic autoimmune diseases and plays a key role in SLE and in SLE-related disorders (38). Although there is ample evidence that thrombosis and fetal loss are complement dependent in experimental models of APS, complement activation in APS patients is still matter of debate (15). Our results show for the first time increased percentages of C4d complement activation product that is stably deposited on cell membranes of platelets and erythrocytes in PAPS.

High percentages of platelet C4d were reported to be specific for SLE (17–24). We confirmed this finding in aPL negative SLE patients included in this study as a pathological control group. The percentages of C4d bound to circulating blood cells, in particular to platelets, display higher sensitivity than the conventional assays that measure soluble complement components and their unstable activation products. Consequently, platelet C4d percentages have been suggested as useful and sensitive tool for mirroring complement activation in SLE patients (17).

The finding of increased percentages of PC4d and EC4d in PAPS and in aPL positive carriers but not in healthy controls and patients with ITP strongly suggests that the presence of aPL is specifically associated with *in vivo* complement activation. Comparable percentages were found in aPL negative and aPL positive SLE patients with no APS clinical manifestations supporting the hypothesis that SLE and the presence of aPL may be independently linked to complement activation. On the other hand, full blown APS was associated with the highest EC4d and PC4d percentages both in PAPS and in SLE-associated APS. Since the majority of these patients had a history of vascular thrombotic events, we investigated whether a previous thrombosis may affect the percentages of C4d positive cells in the absence of aPL. This possibility was ruled out by the observation that the control group of aPL negative thrombotic patients had normal percentages of C4d positive cells further supporting the specificity of this parameter for *in vivo* complement activation in APS.

Although the serum levels of C3 and C4 in PAPS and in aPL positive carriers were reduced in comparison with healthy controls and patients with ITP or previous thrombosis without aPL, they were still within the normal range. Moreover, C4d positive cells significantly correlated with both serum C3 and C4 values and titers of anti-β_2_GPI and β_2_GPI-dependent anti-CL IgG. These findings are consistent with the hypothesis that the presence of aPL is associated with complement activation in APS.

Few recent studies reported reduced complement levels and increased levels of activation products in some APS patients albeit without clear association with the clinical manifestations of the syndrome (12–14). The high percentages of both EC4d and PC4d in our study further support the involvement of complement in APS. The lack of association with the clinical manifestations of the syndrome was confirmed in our study in spite of the use of a more sensitive and reliable laboratory tool for detection of complement activation.

Complement activation in APS patients should be associated with complement deposition in damaged tissues. There is sound evidence that this is the case for placenta both in animals and in patients (15). A role of complement for the aPL-mediated placenta damage has been suggested in animals while it is still matter of research in humans (15). On the other hand, although less studies looked at vessel complement deposition, the available data are suggestive for a role of complement in endothelial perturbation that may be pivotal for the thrombophilic state (16, 39).

Both circulating and tissue immune complexes are known to play a critical role in activating the complement cascade in SLE (38). Their deposition on the membrane of circulating blood cells explains the increased percentages of C4d positive cells; however additional pathways have been suggested to mediate the binding of complement components to activated platelets without the need of immune complex formation (40). In contrast, the mechanism(s) responsible for complement activation in APS has not been clarified yet. Although circulating anti-β_2_GPI/β_2_GPI complexes can be found in a limited proportion of patients (41, 42), they are undetectable in most of them suggesting that tissue bound complexes (e.g., on vessel walls) (16, 39) and/or additional mechanisms play a role in complement activation.

Beta_2_GPI is the main target for diagnostic and pathogenic aPL and it is general accepted that binds to activated platelets through several candidate membrane receptors or just because of the electrostatic interaction between the cationic PL-binding site of β_2_GPI and the anionic phospholipid (i.e., phosphatidylserine) that is exposed in the outer cell membrane leaflet upon cell activation (3). Once bound, the adhered β_2_GPI exposes the immunodominant epitope on D1 that is recognized by pathogenic aPL that in turn may activate complement (3). With this in mind, we set up an *in vitro* model in which normal platelets were incubated with anti-D1 monoclonal antibody in the presence or absence of a platelet agonist (i.e., TRAP) and autologous serum as a source of β_2_GPI and complement. Our *in vitro* results support only in part the role of immune complexes for complement deposition on the platelet cell membrane, because only a small percentage of activated platelets actually displayed membrane C4d. Additional mechanisms that do not require immune complex formation at the platelet surface may play a role as also suggested in SLE patients (40). Moreover, we can speculate that abnormalities in PAPS or SLE platelets themselves may be responsible for the increased complement deposition on their surface.

In conclusion the detection of complement activation products on circulating erythrocytes and platelets using a highly sensitive and specific assay further supports the view that APS is a complement-mediated disorder. Increased EC4d and PC4d percentages are associated with the active inflammatory disease in SLE. It is difficult to translate this finding to APS which is a non-acute inflammatory disorder. We failed to find an association with both the classification and non-classification criteria, including thrombocytopenia. However, we believe that this sensitive tool to evaluate complement activation may offer more information in monitoring the dynamics of the aPL-mediated pathogenic pathways during pregnancy rather than in patients far from the acute thrombotic events.

## Ethics Statement

This study was carried out in accordance with the standard of the good clinical practice with written informed consent from all subjects. All subjects gave written informed consent in accordance with the Declaration of Helsinki. The protocol was approved by the ethical committee “Milano Area 2 426_2014.”

## Author Contributions

PL, MS, and MG wrote the first draft of the manuscript. PL and MS performed the *ex-vivo* and *in-vitro* studies and interpreted data. MG, GP, and WB evaluated and recruited the patients. FP performed statistical analysis. DC detected aPL. EAF contributed to developing the *in-vitro* system. MB, MC, FT, and PM contributed to conception and design of the study. All authors contributed to manuscript revision, read and approved the submitted version.

### Conflict of Interest Statement

The authors declare that the research was conducted in the absence of any commercial or financial relationships that could be construed as a potential conflict of interest.
