# Supplementary material for: Blood Cell-Bound C4d as a Marker of Complement Activation in Patients With the Antiphospholipid Syndrome
Source: Front Immunol. 2019 Apr 12;10:773. doi: 10.3389/fimmu.2019.00773 (PMC6474283; doi:10.3389/fimmu.2019.00773)
Supplement: Supplementary file 1 [file Data_Sheet_1.docx]

**SUPPLEMENTARY MATERIALS**

**1 Supplementary tables and figures**

**1.1 Tables**

**Supplementary Table 1 – Demographic, laboratory and clinical features of enrolled subjects.**

|  | **NHS**  **(n=26)** | **ITP**  **(n= 11)** | **aPL neg**  **thrombosis**  **(n= 8)** | **aPL neg SLE**  **(n= 16)** | **aPL pos SLE**  **(n=17)** | **SAPS**  **(n= 11)** | **aPL pos carriers**  **(n= 8)** | **PAPS**  **(n= 23)** |
| --- | --- | --- | --- | --- | --- | --- | --- | --- |
| M/F | 4/22 | 6/5 | 2/6 | 3/13 | 1/16 | 0/11 | 1/7 | 10/13 |
| Age mean ± SD | 36 ± 7 | 62 ± 19 | 78 ± 20 | 43 ± 16 | 41 ± 13 | 45 ± 14 | 47 ± 11 | 48 ± 12 |
| Thrombotic manifestations^a^ (%) | 0 | 1 (9) | 8 (100) | 1 (6) | 0 | 8 (73) | 0 | 18 (78) |
| Obstetric + thrombotic manifestations^a^ (%) | 0 | 0 | 0 | 0 | 0 | 1 (9) | 0 | 1 (4.3) |
| Obstetric manifestations^a^ (%) | 0 | 0 | 0 | 0 | 0 | 2 (18) | 0 | 4 (17.4) |
| SLEDAI median (min-max) | - | - | - | 4 (0-16) | 4 (0-12) | 2 (0-14) | - | - |
| Thrombocytopenia (%)  Platelets 100-150 x 10^3^/µL  Platelets <100 x 10^3^/ µL | 0 (0)  0 (0)  0 (0) | 11 (100)  1 (9)  10 (91) | 0 (0)  0 (0)  0 (0) | 2 (12.5)  1 (50)  1 (50) | 4 (22)  1 (25)  3 (75) | 4 (36.4)  4 (100)  0 (0) | 1 (12.5)  0  1 (100) | 8 (35)  5 (62.5)  3 (37.5) |
| medium/high aCL IgG (%) | 0 (0) | 0 (0) | 0 (0) | 0 (0) | 8 (47) | 6 (54) | 5 (63) | 20 (87) |
| medium/high aCL IgM(%) | 0 (0) | 0 (0) | 0 (0) | 0 (0) | 3 (17.6) | 0 (0) | 1 (13) | 4 (17) |
| medium/high anti-β_2_GPI IgG (%) | 0 (0) | 0 (0) | 0 (0) | 0 (0) | 4 (24) | 5 (45) | 6 (75) | 19 (83) |
| medium/high anti-β_2_GPI IgM (%) | 0 (0) | 0 (0) | 0 (0) | 0 (0) | 3 (18) | 2 (18) | 3 (38) | 4 (17) |
| LAC (%) | 0 (0) | - | 0 (0) | 0 (0) | 11 (65) | 8 (73) | 4 (50) | 19 (83) |
| Anti-dsDNA (%) | - | - | - | 6 (38) | 4 (24) | 5 (46) | 3 (38) | 0 (0) |
| Anticoagulant therapy^b^ (%) | 0 (0) | 1 (9) | 4 (50) | 0 (0) | 0 (0) | 5 (45) | 1 (12,5) | 15 (65) |
| Aspirin (%) | 0 (0) | 0 (0) | 3 (37.5) | 1 (6) | 8 (47) | 5 (45) | 2 (25) | 12 (52) |
| Hydroxychloroquine (%) | 0 (0) | 0 (0) | 0 (0) | 12 (75) | 15 (88) | 5 (45) | 4 (50) | 11 (48) |
| Prednisone use (%) | 0 (0) | 2 (18) | 0 (0) | 15 (94) | 15 (88.2) | 7 (63.6) | 1 (12.5) | 5 (21.7) |
| Prednisone dose (mg) median  (min-max) | - | 37.5 (25-50) | - | 10 (5-25) | 10 (5-30) | 25 (2.5-25) | 15 | 7.5 (5-7.5) |
| Immunosuppressive therapy (%) | 0 (0) | 0 (0) | 0 (0) | 9 (56) | 10 (59) | 6 (54) | 1 (12.5) | 2 (9) |

NHS, normal healthy subjects; ITP, primary immune thrombocytopenia; aPL, anti-phospholipid antibodies; SLE, systemic lupus erythematosus; SAPS, secondary antiphospholipid syndrome; PAPS, primary antiphospholipid syndrome; aCL, anticardiolipin antibodies; anti-β_2_GPI, anti-β_2_-glycoprotein I antibodies, LAC, lupus anticoagulant.

^a^as defined according to the classification criteria by Miyakis and colleagues (Miyakis S, Lockshin MD, Atsumi T, Branch DW, Brey RL, Cervera R, et al. International consensus statement on an update of the classification criteria for definite antiphospholipid syndrome (APS). Journal of thrombosis and haemostasis : JTH. 2006;4(2):295-306. Epub 2006/01/20).

^b^Vitamin K agonists.

**1.2 Figures**

*
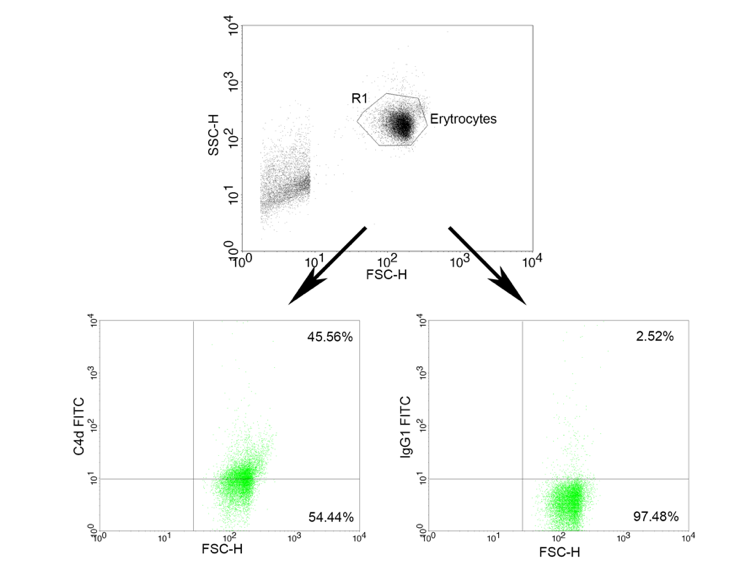
*

**Supplementary Figure 1: Erythrocytes gating strategy**

Forward versus side scatter (FSC vs SSC) gating has been used to identify the erythrocytes in EDTA-whole blood. Cells were stained with mouse anti-human C4d monoclonal antibody or mouse anti-human isotype control IgG1κ, followed by addition of FITC-conjugated goat anti-mouse IgG antibody. 5000 events in the erythrocytes gate were acquired with a high flow rate. A single fluorochrome dot plot strategy (FSC vs FITC) was used for quantification of the percentage of C4d or isotype control positive erythrocytes in R1.


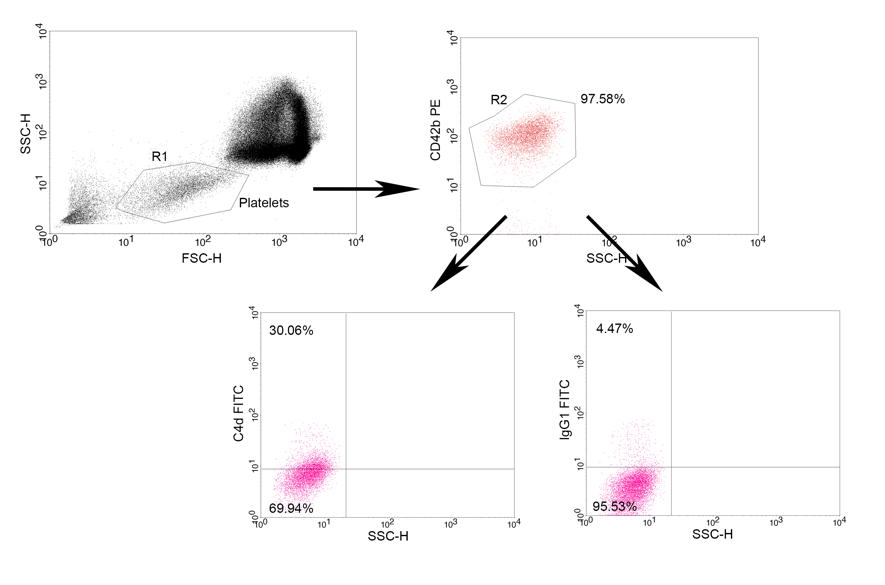


**Supplementary Figure 2: Platelets gating strategy**

Forward versus side scatter (FSC vs SSC) gating has been used to identify the platelets in EDTA-whole blood (R1). Samples were stained with mouse anti-human C4d monoclonal antibody or mouse anti-human isotype control IgG1κ, followed by FITC-conjugated goat anti-mouse IgG antibody. A PE–conjugated monoclonal antibody against human CD42b was used to identify platelets (R2). 5000 events in the platelet gate (R1) were acquired at low flow rate. A single fluorochrome dot plot strategy was used to identify CD42b positive platelets (SSC vs CD42b-PE, R2) and the percentage of C4d or isotype control positive platelets was measured in R2 region (SSC vs FITC).


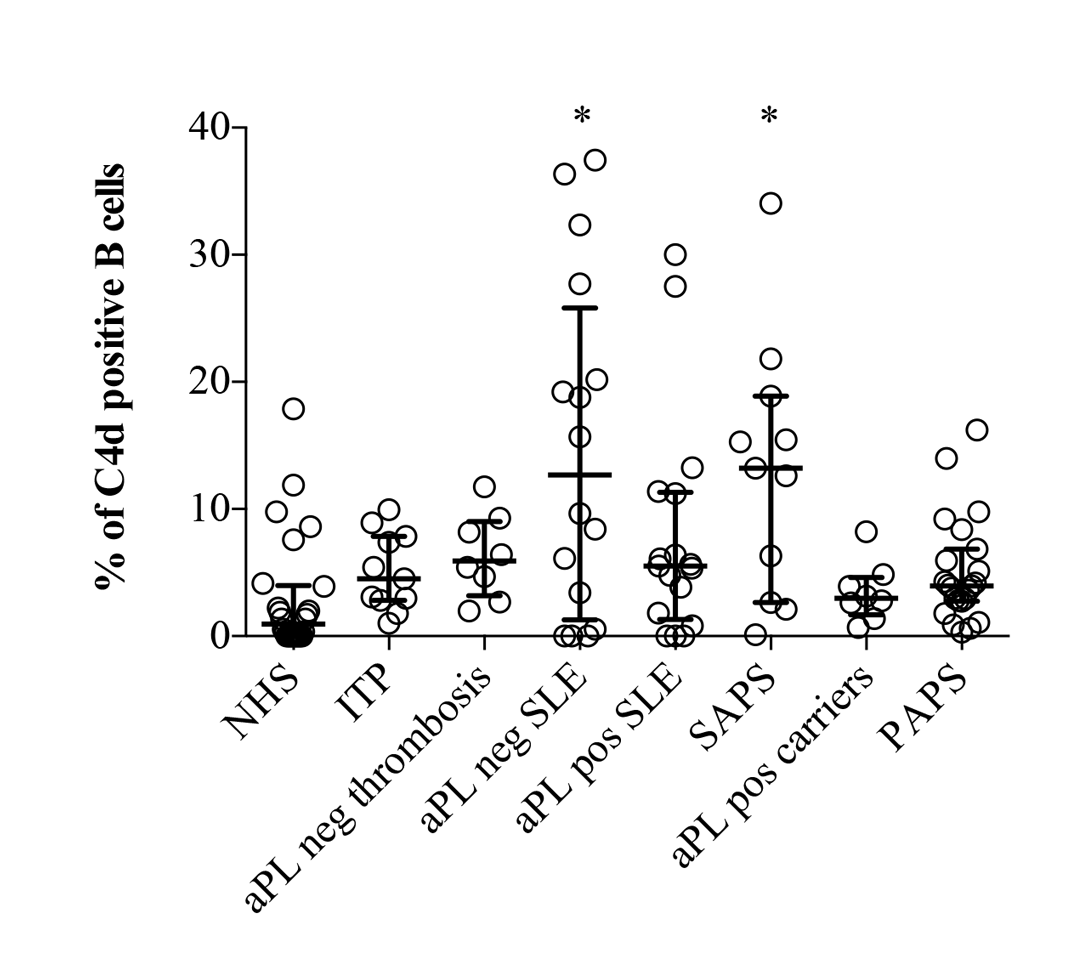


**Supplementary Figure 3: C4d deposition on B lymphocytes**

Flow cytometry was performed on EDTA-whole blood samples (n=120). C4d-positive cells were detected by purified anti-human C4d and FITC-conjugated goat anti-mouse antibody on B lymphocytes. Results are expressed as percentage, median with interquartile range, and analysed by Kruskal-Wallis test and Dunn’s multiple comparison post hoc test. **p*<0.05.

*
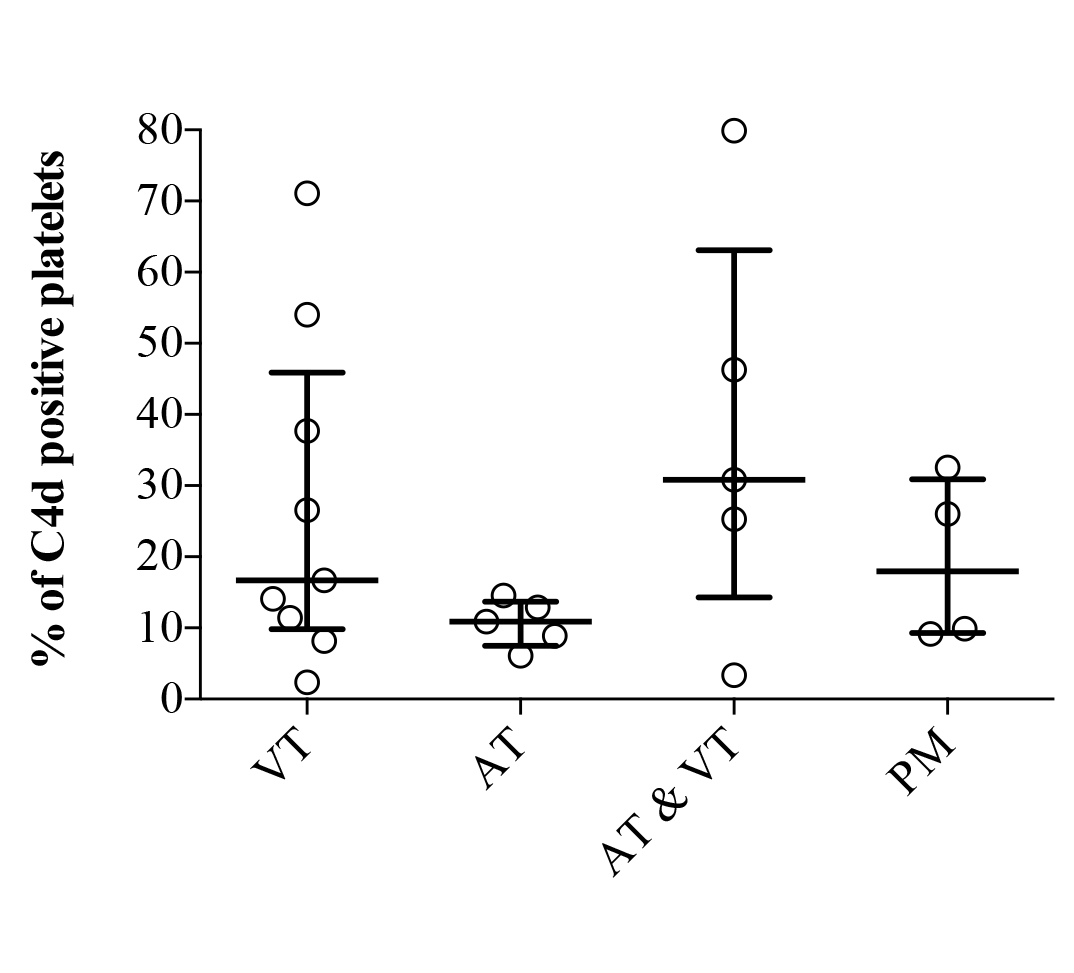
*

**Supplementary Figure 4: PC4d percentages in vascular and obstetric PAPS**

Flow cytometry was performed on EDTA-whole blood samples (n=23) from vascular and obstetric PAPS. C4d-positive platelets were detected by purified anti-human C4d and FITC conjugated goat anti-mouse antibody. Data are expressed as percentage, median with interquartile range, and analysed by Kruskal-Wallis test and Dunn’s multiple comparison post hoc test. VT: venous thrombosis; AT: arterial thrombosis; AT & VT: arterial and venous thrombosis; PM: pregnancy morbidity.


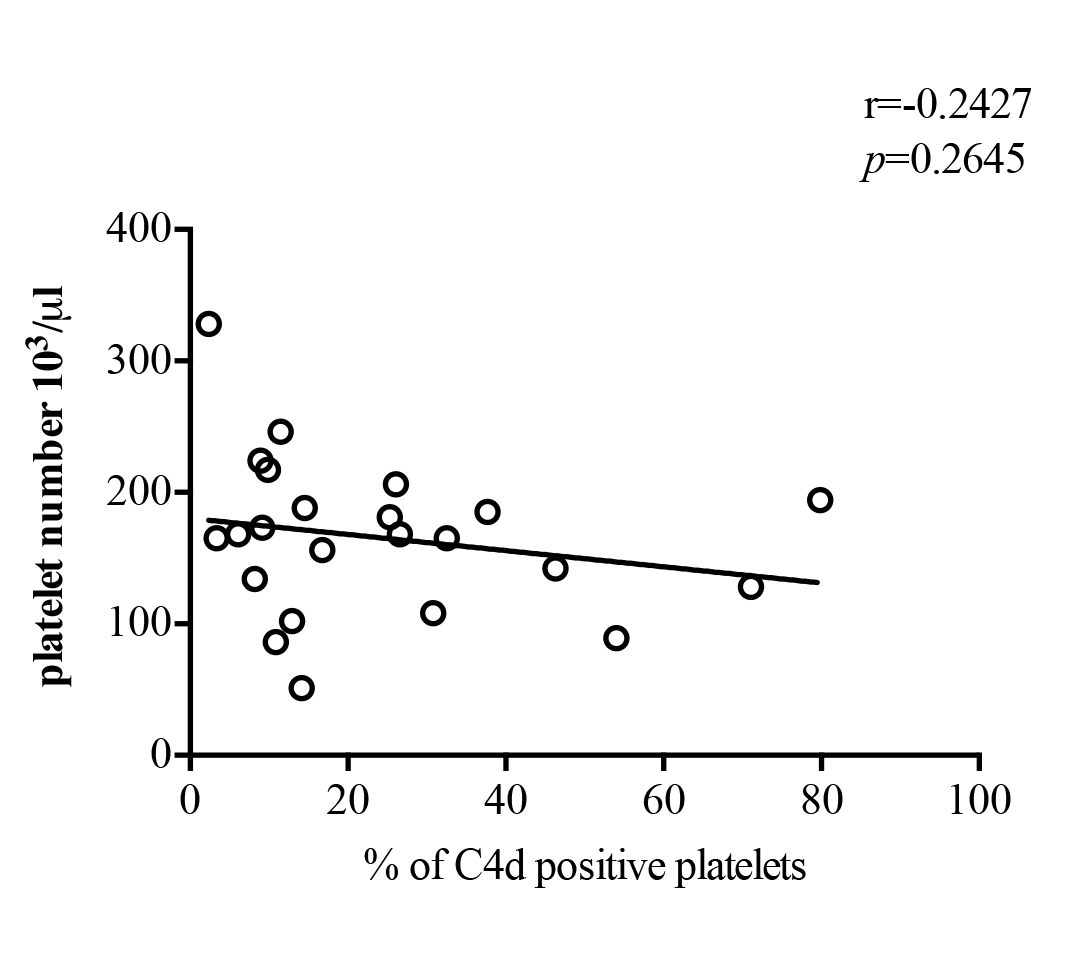


**Supplementary Figure 5: Correlation between platelet number and PC4d percentage**

PC4d percentages from vascular or obstetric PAPS did not correlate with platelet number.

*
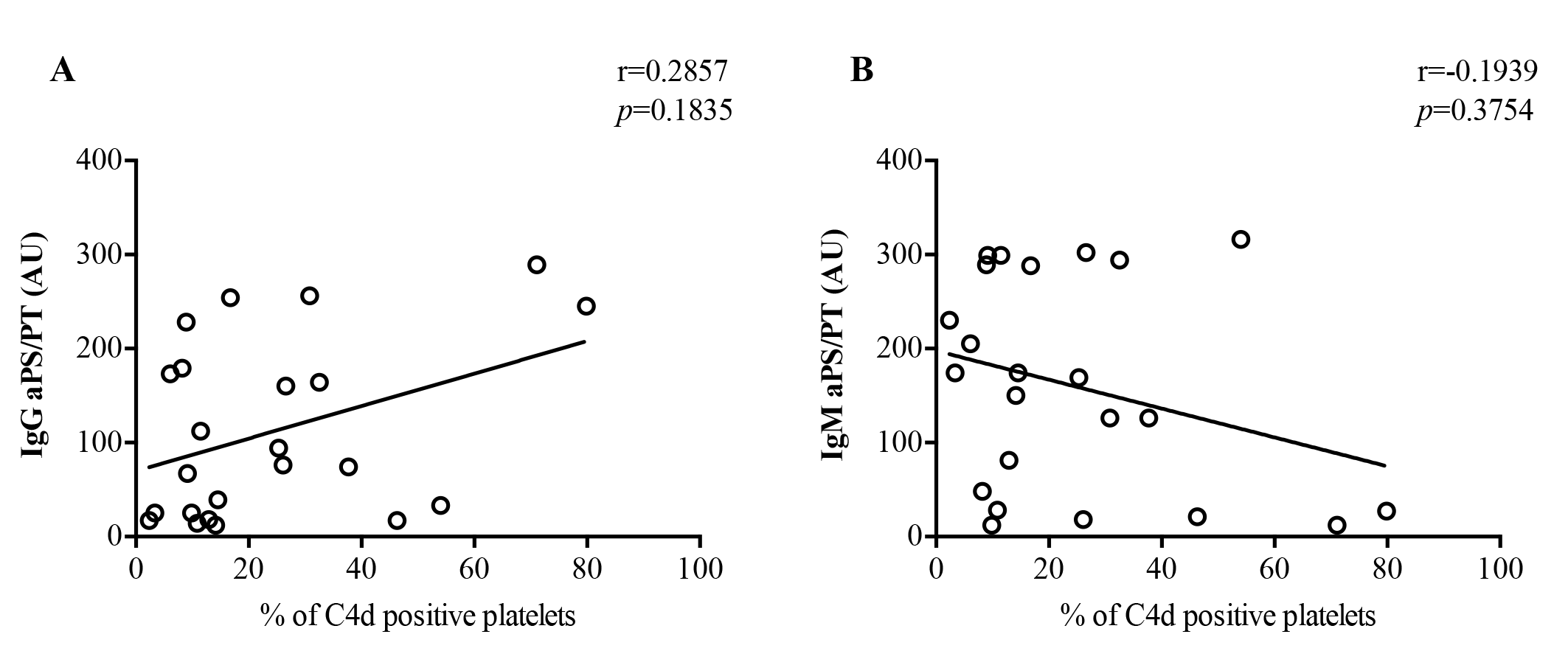
*

**Supplementary Figure 6: Correlations between anti-PS/PT titres and PC4d percentages.**

PC4d percentages did not correlate with IgG anti-PS/PT **(A)**, nor with IgM anti-PS/PT **(B)**. Anti-PS/PT IgG and IgM have been detected as previously described (Pregnolato F. et al., Immunol. Res., 2013 Jul;56(2-3):432-8)

*
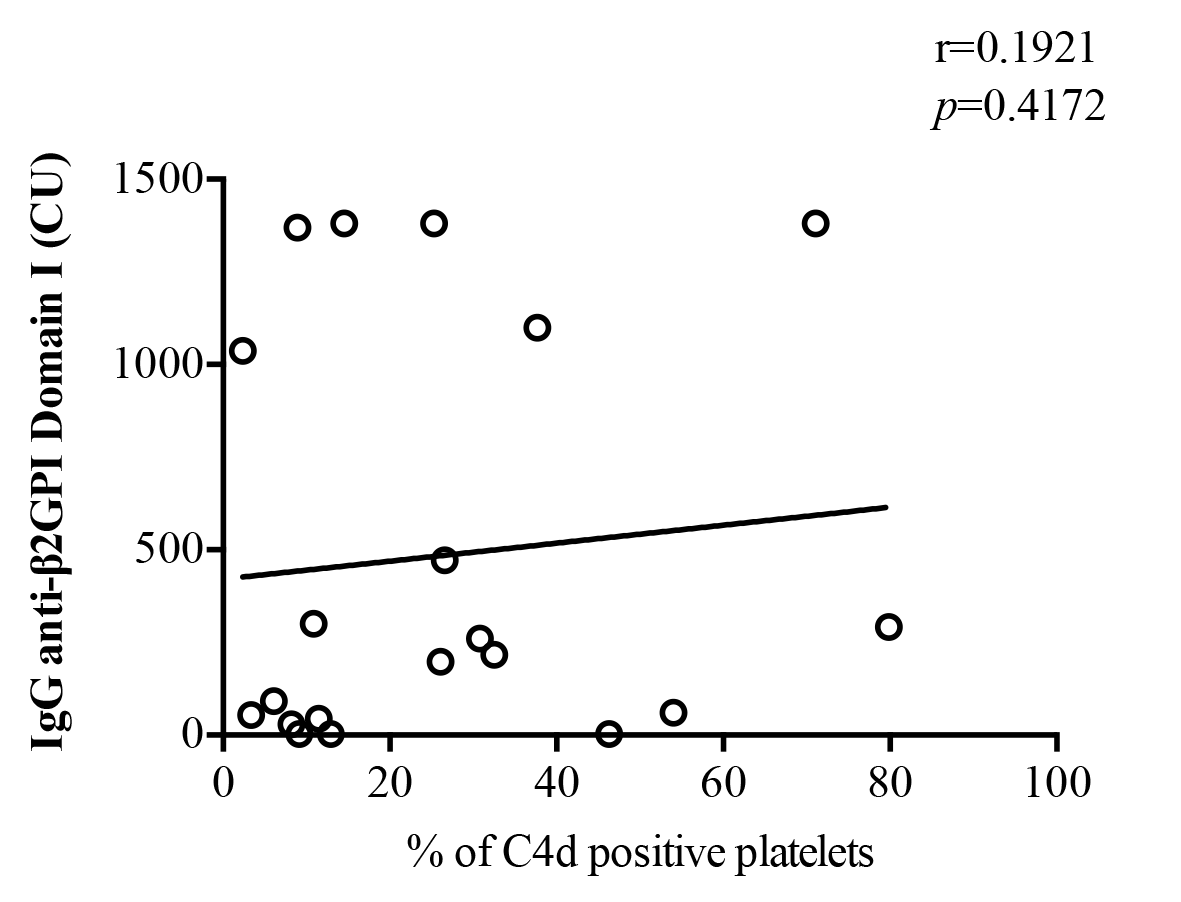
*

**Supplementary Figure 7: Correlations between IgG anti-β_2_GPI Domain I titres and PAPS PC4d percentages.**

PC4d percentages did not correlate with IgG anti-β_2_GPI Domain I titres. Anti-domain 1 IgG have been detected as previously described (Durigutto P. et al., Haematologica., 2018, Nov 15, pii: haematol.2018.198119 [Epub ahead of print])

*
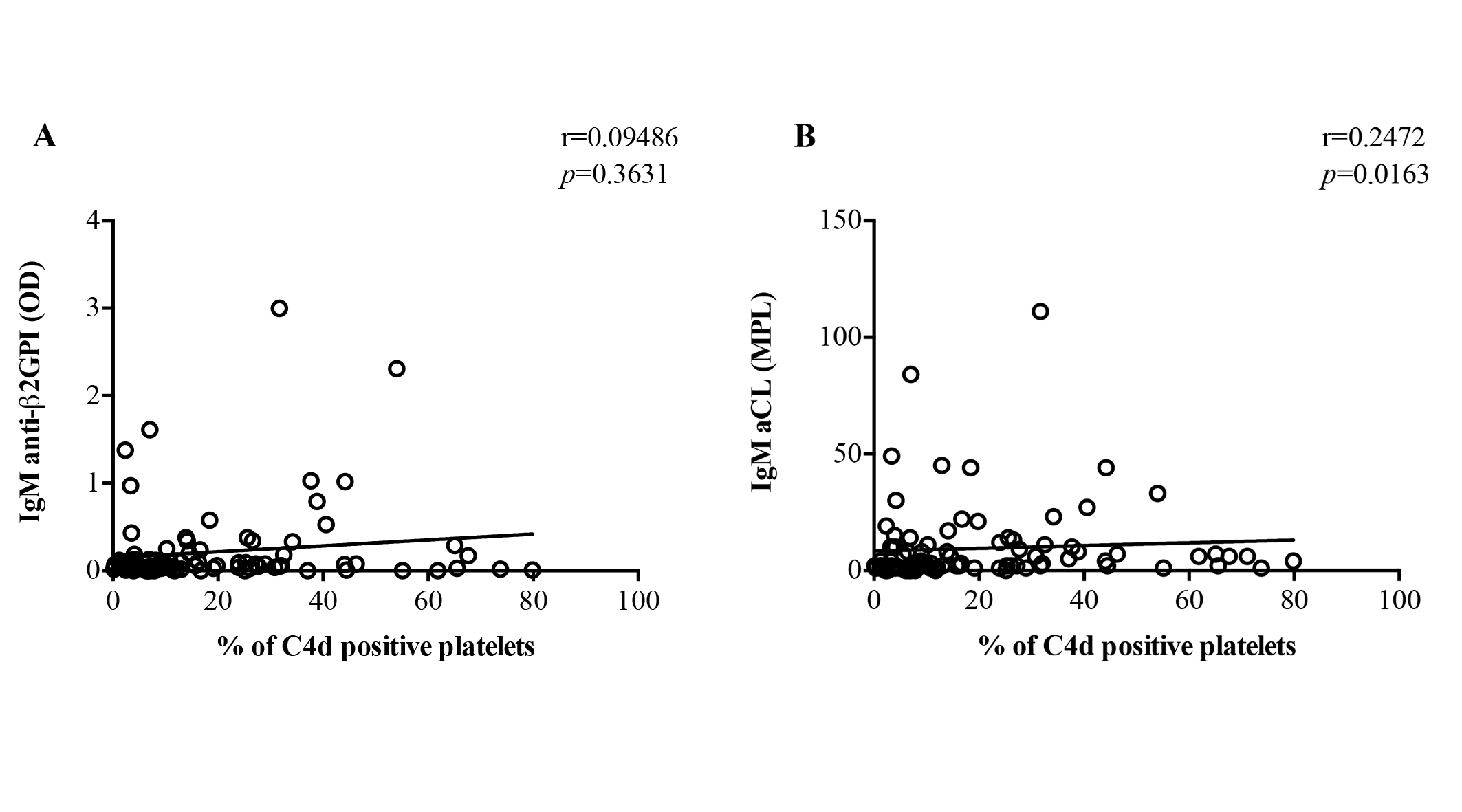
*

**Supplementary Figure 8: Correlations between PC4d percentages and anti-β_2_GPI or anti-CL IgM titres**

PC4d percentages did not correlate with anti-β_2_GPI IgM **(A)** while correlated with a-CL IgM **(B)** titres.


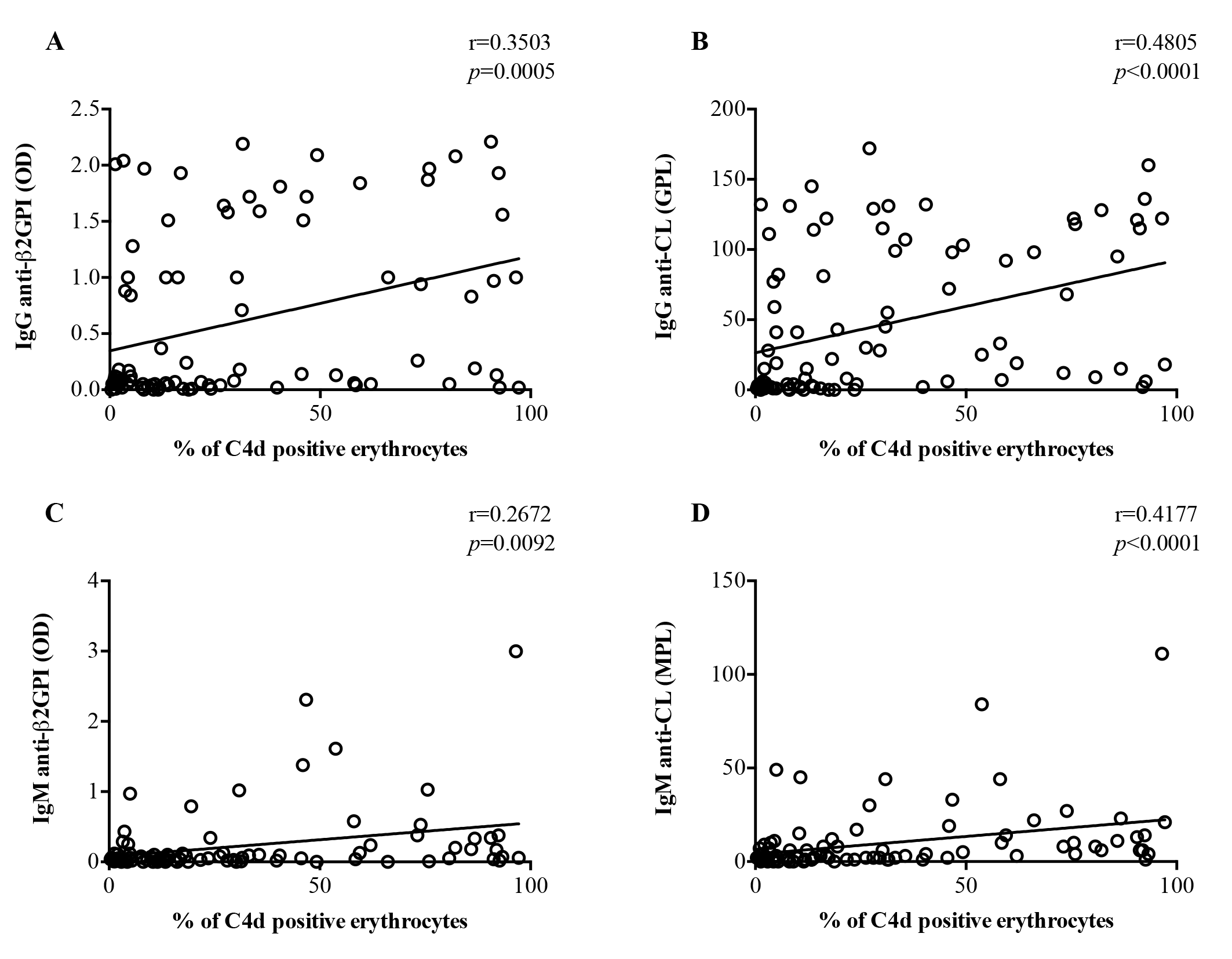


**Supplementary Figure 9: Correlations between EC4d percentages and anti-β_2_GPI or anti-CL titres**

EC4d percentages positively correlated with IgG and IgM anti-β_2_GPI **(A, C)** and IgG and IgM anti-CL **(B, D)** titres.
